# Hemodynamic monitoring and management in patients undergoing high risk surgery: a survey among North American and European anesthesiologists

**DOI:** 10.1186/cc10364

**Published:** 2011-08-15

**Authors:** Maxime Cannesson, Gunther Pestel, Cameron Ricks, Andreas Hoeft, Azriel Perel

**Affiliations:** 1 Department of Anesthesiology and Perioperative Care, School of Medicine, University of California, Irvine, 101 S City Drive, Orange, CA 92868, USA; 2 Department of Anesthesiology, Johannes Gutenberg-University Medical Center, Langebeckstraβe 1, 55131 Mainz, Germany; 3 Department of Anesthesiology and Intensive Care Medicine, University Hospital Bonn, Sigmund Freu Straβe 25, 53127 Bonn, Germany; 4 Department of Anesthesiology and Intensive Care, Sheba Medical Center, Tel Aviv University, Ramat Gan 52621, Tel Aviv, Israel

## Abstract

**Introduction:**

Several studies have demonstrated that perioperative hemodynamic optimization has the ability to improve postoperative outcome in high-risk surgical patients. All of these studies aimed at optimizing cardiac output and/or oxygen delivery in the perioperative period. We conducted a survey with the American Society of Anesthesiologists (ASA) and the European Society of Anaesthesiology (ESA) to assess current hemodynamic management practices in patients undergoing high-risk surgery in Europe and in the United States.

**Methods:**

A survey including 33 specific questions was emailed to 2,500 randomly selected active members of the ASA and to active ESA members.

**Results:**

Overall, 368 questionnaires were completed, 57.1% from ASA and 42.9% from ESA members. Cardiac output is monitored by only 34% of ASA and ESA respondents (*P *= 0.49) while central venous pressure is monitored by 73% of ASA respondents and 84% of ESA respondents (*P *< 0.01). Specifically, the pulmonary artery catheter is being used much more frequently in the US than in Europe in the setup of high-risk surgery (85.1% vs. 55.3% respectively, *P *< 0.001). Clinical experience, blood pressure, central venous pressure, and urine output are the most widely indicators of volume expansion. Finally, 86.5% of ASA respondents and 98.1% of ESA respondents believe that their current hemodynamic management could be improved.

**Conclusions:**

In conclusion, these results point to a considerable gap between the accumulating evidence about the benefits of perioperative hemodynamic optimization and the available technologies that may facilitate its clinical implementation, and clinical practices in both Europe and the United States.

## Introduction

Mortality and morbidity related to anesthesiology have significantly decreased during the last decade, mainly due to improvements in patients' safety in general, as well as better risk stratification and better management. However, complications following major surgery are still a leading cause of perioperative morbidity and mortality [1,2]. High-risk surgical patients represent only about 10% of the overall anesthesiology procedures performed each year, and yet these patients account for over 80% of perioperative deaths [3]. This represents a substantial global public-health concern since it is estimated that 234 million major surgical procedures are performed worldwide each year including 40 millions in the US alone [4].

Several studies have demonstrated that perioperative hemodynamic optimization has the ability to improve postoperative outcome in high-risk surgical patients [5-7]. Specifically, hemodynamic optimization in patients undergoing high-risk surgery has been shown to decrease the incidence of postoperative complications, to decrease length of stay in the intensive care unit and in the hospital, to decrease the overall cost of surgery [6], and to possibly improve long-term survival [8]. More than a decade ago it was already claimed that it may be considered unethical not to use goal-directed perioperative therapy once patient identification and the methods to be used in treating them are refined [9]. And yet, the principles of perioperative optimization are not applied uniformly, if at all, and there is a great variability in their adoption into clinical practice.

Part of the observed non-uniformity in the clinical application of perioperative optimization may be due to the prevalent different practices in hemodynamic monitoring with regard mainly to the measurement of cardiac output (CO). The most frequent parameters that have been used for perioperative optimization are cardiac output (CO) and/or oxygen delivery (DO2). Although the proclaimed gold standard for cardiac output measurement is still intermittent thermodilution by the pulmonary artery catheter (PAC), the use of this device has dramatically decreased in surgical patients over the past 15 years [10]. This decrease is mostly related to the fact that the PAC is highly invasive and has several associated risks [10]. One should consider, however, that the apparent decline in the use of PACs is due to both a shift in philosophy and its replacement by new technologies [11].

The aim of the present study is, therefore, to assess and report current hemodynamic management practices in patients undergoing high-risk surgery in Europe and in the United States using a self reported internet survey. The results from this study will help to determine the potential need for future educational endeavors and practice guidelines regarding hemodynamic monitoring and management in patients undergoing high-risk surgery.

## Materials and methods

A survey of 33 questions was developed to assess the current trend in hemodynamic management and monitoring for high-risk surgery patients. Twelve questions were related to the respondents' demographic data and practice.

The Institutional Review Board at the University of California Irvine approved the study. An invitation to participate in the survey was emailed through the American Society of Anesthesiology (ASA) e-newsletter to 2,500 randomly selected active members of the ASA, representing approximately 10% of the active membership. At the same time, an email was sent through the European Society of Anaesthesiology (ESA) office to active ESA members with a link to the survey and a link to the survey was posted on the European Society of Anaesthesiology website. Below is the invitation that was sent to ASA and ESA members:

Dear ASA/ESA member,

We are inviting you to participate in a research project regarding our current practices of hemodynamic monitoring and management in patients undergoing high-risk surgery. Information regarding your practice, experiences, philosophies, and training will be used to assess the potential need for future educational endeavors and practice guidelines.

The way in which patients are monitored and their outcomes are optimized is rapidly evolving and improving. However, setting a standard of care with the use of new methods has yet to be achieved. In particular, hemodynamic monitors and indices available in high-risk surgery patients have markedly evolved. Currently, even if pulmonary artery catheterization remains the gold standard for hemodynamic monitoring in high-risk patients, its use has dramatically declined (63%) in the last 15 years. While this decline is largely due to the fact that the device is highly invasive and has several associated risks, it may also be due to changes in philosophy or implementation of new technologies.

This questionnaire has been approved by the ASA/ESA for distribution to its members. At the end of this email there is a link to a 33-question Survey Monkey questionnaire, which should take about 10 minutes to complete. Participation in this questionnaire is completely voluntary and anonymous. We hope that you will take the time to fill out this questionnaire and help us to uncover the potential need for standardizing hemodynamic practices in high-risk surgical patients.

Resident, affiliate, honorary, life, and retired members were excluded. A covering letter explained that the data collected would be anonymous and be non-attributable. Participants accessed a Web site linked to a secure database (Survey Monkey, Palo Alto, CA, USA). The survey was opened from 1 October 2010 to 31 December 2011. To maximize response rate, we sent two sequential e-mails to the participants during the study period.

### Survey questions

In the survey, high risk surgery patients were defined and presented according to the definition presented below:

For the following questionnaire, we will define high risk surgery patients as patients aged 18 years or older presenting for major surgery expected to last more than 1.5 hours and having at least two of the following criteria:

1. Cardiac or respiratory illness resulting in functional limitation

2. Extensive surgery planned for carcinoma involving bowel anastomosis

3. Predictable acute massive blood loss (> 2.5 liters)

4. Aged over 70 years with functional limitation of one or more organ systems

5. Septicemia (positive blood cultures or septic focus)

6. Respiratory failure (PaO2 < 8 kPa on FiO2 > 0.4, that is, PaO2:FiO2 ratio < 20 kPa or ventilation > 48 hours)

7. Acute abdominal catastrophe (for example, pancreatitis, perforated viscous, gastro-intestinal bleed)

8. Acute renal failure (urea > 20 mmol l^-1^, creatinine > 260 μmol l^-1^)

9. Surgery for abdominal aortic aneurysm

10. Disseminated malignancy

This definition has been used in previously published studies on the topic both in the US and in Europe [5,12,13]. The full questionnaires sent to ASA and ESA members are presented in Appendix 1 and 2 respectively.

### Statistical analysis

Categorical data are expressed as frequency. Data were analyzed according to the number of responses we obtain for each given question. Categorical items were analyzed by frequency distribution and χ2 analysis. In all cases, two-tailed *P*-values of 0.05 or less were considered evidence of differences not attributable to chance. All analyses were performed using SPSS 11.0 (SPSS Inc., Chicago, IL, USA).

## Results

### Respondents' description

We received 217 responses from ESA members and 273 responses from ASA members. Total completed questionnaires were 158 (72.8%) for ESA members and 210 (76.9%) for ASA members. Overall, 368 questionnaires were completed, including 57.1% from ASA members and 42.9% from ASA members.

#### ASA respondents' descriptions

ASA respondents are working mostly (48%) in private practice with lesser numbers working in general hospitals (24.2%) and in university hospitals (25.4%). About half (51.8%) of them take care of high risk surgery patients 1 to 5 times a week and about a third (35.1%) of them do it 6 to 10 times a week, with only 13.8% of them taking care of cardiac surgery patients. Half of them had further training, including a fellowship in cardiac anesthesia (48.5%) and in critical care (25.8%). About a third (33.8%) finished their training after 2000 and 6.2% before 1980. Only 31.6% of the ASA respondents manage high-risk surgical patients in the intensive care unit.

#### ESA respondents description

ESA respondents are working mostly (54.0%) in university hospitals, with lesser numbers working in general hospitals (37.6%) and in private practice (5.0%). The majority, (72.8%), take care of high-risk surgery patients 1 to 5 times a week and 18.3% do it 6 to 10 times a week, with 10.3% taking care of cardiac surgery patients. One third (31.2%) of ESA member respondents had further training, including a fellowship in critical care (58.3%) or in cardiac anesthesia (33.3%). About half (45.8%) finished their training after 2000 and 7.1% before 1980. The majority (79.7%) of ESA respondents manage high-risk surgical patients in the intensive care unit.

**Figure 1:**
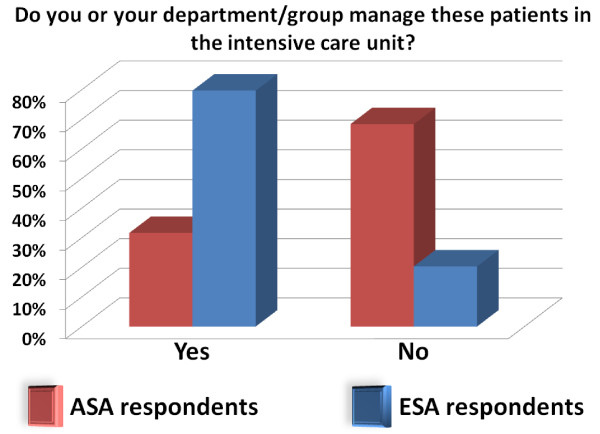
Do you or your department/group manage these patients in the intensive care unit?

Although more ASA respondents work in private practice than ESA respondents (48.0% vs. 5.0%; *P *< 0.001), they manage high-risk surgery patients more often than ESA respondents (6 to 10 times/week in 35.1% of ASA respondents vs. 18.7% of ESA respondents; *P *< 0.001). Interestingly, 18.6% of ESA respondents work in hospitals of > 1,000 beds (vs. 5.7% ASA respondents; *P *< 0.001); yet intensive care units with > 40 beds are more frequent in the US (37.9% ASA vs. 17.8% ESA; *P *< 0.001). For ESA respondents, critical care and anesthesiology belong to the same group; this is different for ASA respondents (Figure 1). ESA respondents seem to be younger: 46.2% of them finished training after 2000 (vs. 33.8% of ASA respondents; *P *< 0.001). The term "fellowship" seems unfamiliar to ESA respondents: 24.1% of them answer "not applicable" (vs. 0% of ASA respondents).

### Hemodynamic monitoring and management practices

**Figure 2:**
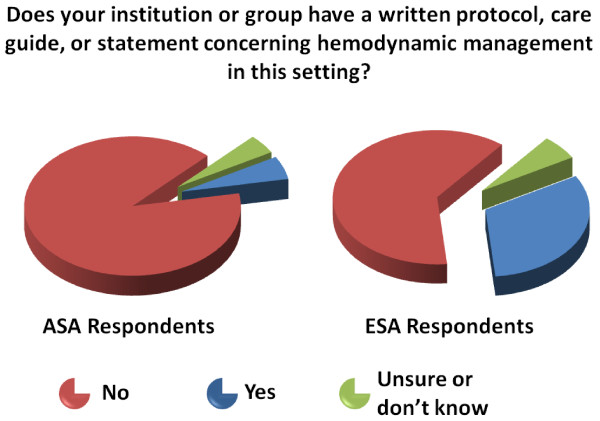
Incidence of institutional guidelines concerning hemodynamic management in this setting?

Hemodynamic monitoring practices seem to differ considerably between Europe and the USA (Table 1), with more Europeans having written protocols concerning hemodynamic management in high-risk surgical patients than Americans (30.4% and 5.4%, respectively, *P *< 0.001) (Figure 2).

**Table 1:** Hemodynamic monitoring used for the management of high-risk surgery patients?.

	ASA respondents (*n *= 237)	ESA respondents (*n *= 195)
**Answer options**	**Response percent**	**Response percent**

Invasive arterial pressure	95.4%	89.7%
Central venous pressure	72.6%	83.6%
Non-invasive arterial pressure	51.9%	53.8%
Cardiac output	35.4%	34.9%
Pulmonary capillary wedge pressure	30.8%	14.4%
Transesophageal echocardiography	28.3%	19.0%
Systolic pressure variation	20.3%	23.6%
Plethysmographic waveform variation	17.3%	17.9%
Pulse pressure variation	15.2%	25.6%
Mixed venous saturation (ScvO2)	14.3%	15.9%
Central venous saturation (SvO2)	12.7%	33.3%
Oxygen delivery (DO2)	6.3%	14.4%
Stroke volume variation	6.3%	21.5%
Near infrared spectroscopy	4.6%	5.1%
Global end diastolic volume	2.1%	8.2%

ASA, American society of anesthesiology respondents; ESA, European society of anaesthesiology respondents.

#### Invasive blood pressure and central venous pressure monitoring

**Figure 3:**
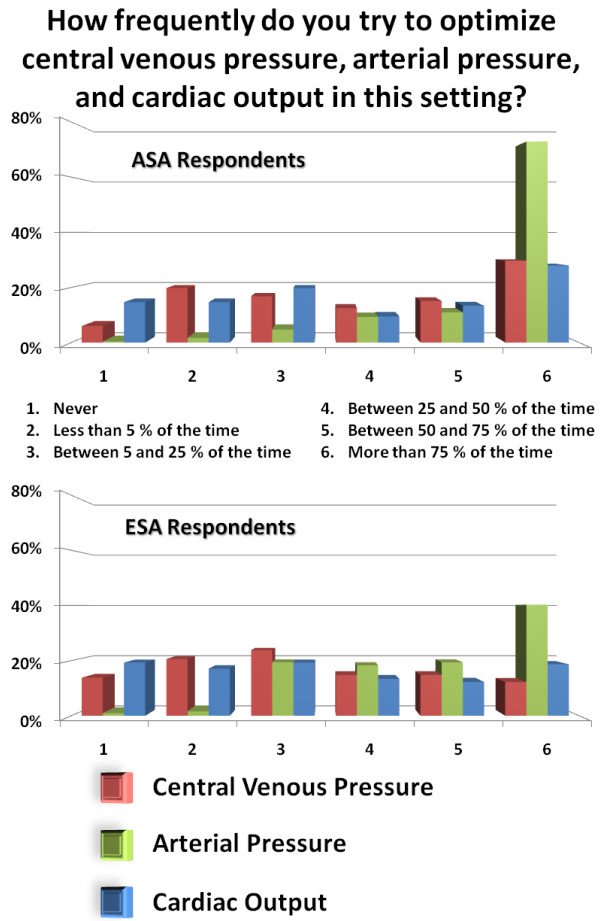
How frequently do you try to optimize central venous pressure, arterial pressure, and cardiac output in this setting?

While invasive arterial pressure is monitored and used for hemodynamic optimization by more than 90% of both ASA and ESA respondents, there seems to exist significant heterogeneity in the way CVP and CO are being monitored and used (Figure 3). Interestingly, CVP is monitored by 84% of ESA respondents and by 73% of ASA respondents (*P *< 0.05). In contrast, ESA or ASA respondents rarely optimize CVP while most of them optimize arterial pressure.

#### Cardiac output monitoring

**Figure 4:**
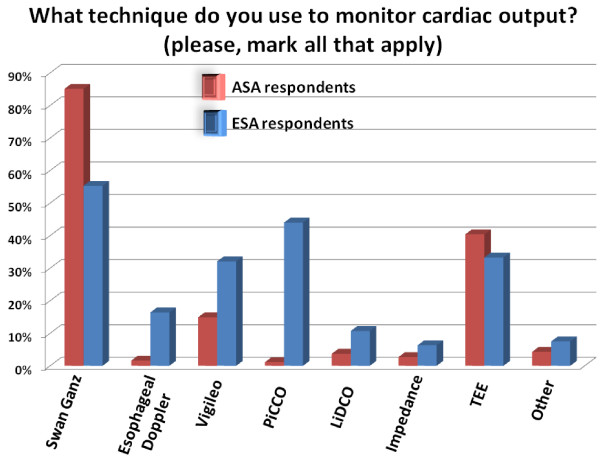
**What technique do you use to monitor cardiac output? (Please, mark all that apply)**.

Although CO is being monitored by only about 34% of both ASA and ESA respondents (*P *= 0.491), we observed significant differences regarding the techniques used for CO monitoring by ASA and ESA respondents (Figure 4). Specifically, the PAC is being used much more frequently in the US than in Europe in the setup of high-risk surgery (85.1% vs. 55.3% respectively, *P *< 0.001). On the other hand, the PiCCO monitor is used in this setup by 44.0% of ESA respondents and by only 1.1% of ASA respondents (*P *< 0.001). When respondents reported that they do not monitor CO, the main reason given by both groups was that they monitor dynamic parameters of fluid responsiveness as surrogates for CO monitoring (Table 2). Additionally, technologies for CO monitoring are considered by the non-users of both groups to be too invasive (Table 2). Interestingly, although CO is being measured by only a third of ASA and ESA respondents, nearly all respondents agree that oxygen delivery is of major importance for patients undergoing high-risk surgery, with more than 90% exhibiting the knowledge that CO is a major determinant of oxygen delivery.

**Table 2:** Main reasons for not monitoring cardiac output?

	ASA Respondents (*n *= 157)	ESA Respondents (*n *= 142)
**Answer Options**	**Response Percent**	**Response Percent**

I use dynamic parameters of fluid responsiveness (Pulse Pressure Variations, Systolic Pressure Variations, Plethysmographic Waveform Variations) as surrogates for cardiac output monitoring	54.1%	60.6%
Available cardiac output monitoring solutions are too invasive	48.4%	26.8%
Cardiac output monitoring does not provide any additional clinically relevant information in this setting	24.2%	14.1%
I use SvO2 and/or ScVO2 as surrogates for cardiac output monitoring	13.4%	26.1%
Available cardiac output monitoring solutions are unreliable	8.3%	15.5%

ASA, American society of anesthesiology respondents; ESA, European society of anaesthesiology respondents.

#### Dynamic parameters of fluid responsiveness

Parameters considered by both ASA and ESA respondents as indicators for volume expansion are shown in Table 3. Clinical experience, blood pressure, CVP, and urine output are the most widely seen indicators of volume expansion. ESA respondents are more likely to use dynamic parameters of fluid responsiveness based on arterial pressure analysis than ASA respondents. The way ASA and ESA respondents assess the hemodynamic effects of a volume expansion is shown in Table 4. Parameters considered as the best predictors of fluid responsiveness by ASA and ESA respondents are shown in Table 5.

**Table 3:** What are your indicators for volume expansion in this setting (diagnostic tools)?

	ASA Respondents (*n *= 209)	ESA Respondents (*n *= 165)
**Answer Options**	**Response Percent**	**Response Percent**

Blood pressure	88.5%	77.6%
Urine output	83.3%	77.0%
Clinical experience	77.5%	64.8%
Central venous pressure	70.8%	64.2%
Cardiac output	49.3%	53.3%
Pulse Pressure Variation or Systolic Pressure Variation	45.0%	55.8%
Transesophageal echocardiography	43.5%	28.5%
Pulmonary capillary wedge pressure	38.8%	24.2%
Plethysmographic Waveform Variation	25.4%	25.5%
Stroke Volume Variation	19.1%	36.4%
Mixed venous saturation (ScvO2)	18.7%	21.8%
Global end diastolic volume	10.5%	17.0%
Central venous saturation (SvO2)	10.0%	34.5%

ASA, American society of anesthesiology respondents; ESA, European society of anaesthesiology respondents.

**Table 4:** How do you routinely assess the hemodynamic effects of volume expansion?

	ASA respondents (*n *= 203)	ESA respondents (*n *= 162)
**Answer options**	**Response percent**	**Response percent**

Increase in blood pressure	92.1%	75.3%
Increase in urine output	84.7%	73.5%
Decrease in heart rate	74.4%	75.3%
Increase in cardiac output	59.1%	54.3%
Decrease in pulse pressure variation or systolic pressure variation	56.7%	54.9%
Decrease in plethysmographic waveform variation	28.6%	25.9%
Increase in mixed venous saturation (SvO2)	22.2%	18.5%
Decrease in stroke volume variation	21.7%	35.2%
Increase in central venous saturation (SvO2)	19.2%	27.8%

ASA, American society of anesthesiology respondents; ESA, European society of anaesthesiology respondents.

**Table 5:** In your opinion, what best predicts an increase in cardiac output following volume expansion?

	ASA Respondents (*n *= 190)	ESA Respondents (*n *= 158)
**Answer options**	**Response percent**	**Response percent**

Transesophageal echocardiography	26.8%	17.7%
Cardiac output	21.1%	20.9%
Blood pressure	14.2%	5.7%
Pulse pressure variation or systolic pressure variation	12.1%	12.0%
Mixed venous saturation (ScvO2)	7.9%	5.7%
Stroke volume variation	5.8%	21.5%
Clinical experience	5.3%	3.2%
Pulmonary capillary wedge pressure	2.1%	3.2%
Central venous saturation (SvO2)	2.1%	1.9%
Central venous pressure	1.1%	3.2%
Global end diastolic volume	1.1%	3.8%
Plethysmographic waveform variations	0.5%	1.3%

ASA, American society of anesthesiology respondents; ESA, European society of anesthesiology respondents.

Respiratory variations in the plethysmographic waveform are being used by 25% of ASA and ESA respondents. Respiratory variations in arterial pressure are eyeballed by 90.3% of ASA respondents compared with 68.0% of ESA respondents (*P *< 0.001), manually calculated by 9.7% of ASA respondents and by 20.6% of ESA respondents, and monitored using specific software by 5.1% of ASA respondents versus 22.9% of ESA respondents (*P *< 0.001). These parameters are optimized more than 50% of the time by 31.2% of ASA respondents and by 28.4% of ESA respondents (*P *< 0.298).

**Figure 5:**
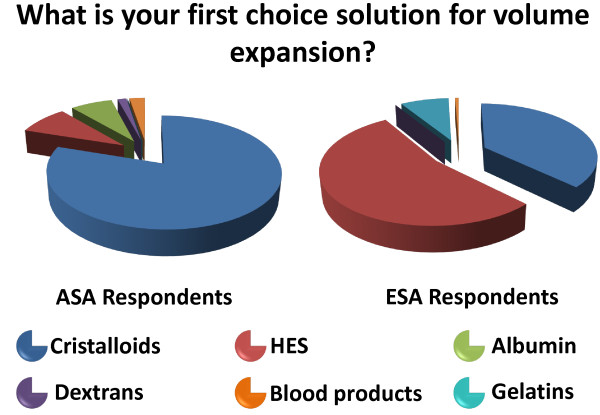
What is your first choice solution for volume expansion?

A significant difference was observed regarding the type of fluid used by ASA and ESA respondents. Crystalloid is the first line therapy used by ASA respondents while ESA respondents chose starches (Figure 5).

To the question: "Do you believe that your current hemodynamic management could be improved?" 86.5% of ASA respondents and 98.1% of ESA respondents (*P *< 0.001) answered "Yes".

## Discussion

The main finding of this international survey is that despite evidence showing that CO optimization during high risk surgery has the potential to improve postoperative patient outcome, and despite the fact that nearly all respondents agreed that oxygen delivery is of major importance for patients undergoing high-risk surgery, only 34% of anesthesiologists in Europe and in the US monitor CO in this setting. More importantly, even those who monitor CO rarely use its value for perioperative optimization as recommended in recent articles [6,8]. Interestingly, 86.5% of ASA respondents and 98.1% of ASA respondents believe that their current hemodynamic management could be improved. The other findings of this survey point out significant differences in the choice and interpretation of monitoring techniques between American and European anesthesiologists

The role of CO monitoring and optimization in improving outcome in high-risk surgery is being repeatedly demonstrated in recent years [6,7,14]. Yet, as our study clearly demonstrates, the adoption of this therapeutic approach is far from being universal. This is reflected by the fact that most of the responding centers do not have clear recommendations or guidelines for hemodynamic management of high-risk surgery patients (Figure 1). One of the main reasons for the apparent reluctance to monitor CO is the belief that in and by itself CO is not an important parameter, and that the clinicians may be better off by using other parameters, like dynamic predictors of fluid responsiveness, as surrogates of CO (Table 2) [15]. However, only 24.2% of ASA respondents and 14.1% of ESA respondents think that CO monitoring does not provide any additional clinically relevant information for the management of high-risk surgery patients. A much larger portion of the respondents, 48.4% of the ASA and 26.8% of the ESA, do not monitor CO because they feel that available solutions are too invasive. Another explanation for the relative scarcity of CO monitoring, which was not explored in our study, is the uncertainty of anesthesiologists about the value of perioperative optimization in general, in view of the studies that have shown that fluid restriction might be beneficial for high-risk surgical patients [16,17].

More than 50% of respondents stated that they are using dynamic predictors of fluid responsiveness as a surrogate for CO monitoring. The ability of dynamic parameters to predict fluid responsiveness accurately has been extensively demonstrated [18-21]. Indeed, functional hemodynamic parameters may be helpful in identifying fluid responders prior to hemodynamic optimization, and, more importantly, identify those patients who are not likely to respond to fluids and, thereby, prevent detrimental fluid overload. However, studies showing an impact of hemodynamic management based on these parameters on patients' outcome are still lacking and so far we have no strong evidence supporting this hypothesis [22]. Moreover, it has been shown recently that about 60% of patients present at least one limitation to the use of dynamic parameters in the operating room [23]. Finally, about 90% of ASA respondents and 70% of ESA respondents only eyeball these variations precluding any strong clinical application.

Our results show that there are significant differences between American and European anesthesiologists in the practice of hemodynamic monitoring. It seems that most clinicians in the US still associate CO monitoring with the use of the Swan-Ganz catheter (Figure 4) while other less invasive monitors are not widely known or used. For example, the transpulmonary thermodilution method that is applied in the PiCCO^® ^and is used by 44.0% of the ESA respondents, seems to be virtually unknown to most clinicians in the US. This could be explained by the fact that this technology requires a femoral arterial line, which is not the standard of care in the US [24]. Another explanation is that technologies seem to spread most in the countries and regions where they are developed and manufactured, due most probably to the involvement of local opinion leaders in the development and validation phases, as well as in the marketing efforts, but this has to be demonstrated. The observed differences between European and US practices in terms of technology and hemodynamic know-how may also stem from the fact that anesthesiologists in Europe are more likely to be ICU and because they manage patients in the ICU more frequently than their American colleagues (Figure 2). We postulate that this may also explain our finding that the number of ESA respondents who use the mixed venous oxygen saturation (SvO2) and/or central venous oxygen saturation (ScVO2) as surrogates for CO monitoring is double than that of ASA respondents since goal directed therapy based on ScvO2 optimization has been validated in septic patients in the intensive care unit [25].

An intriguing finding of our study relates to the monitoring of central venous pressure (CVP) which is still frequently monitored on both continents (Table 1) despite consistent evidence that filling pressures are unreliable in predicting fluid responsiveness [26,27] and that they have numerous limitations [28]. Since most clinicians who monitor CVP admit that they do not try to optimize it (Figure 3), it is unclear how CVP is integrated into the physicians' clinical decision-making process. In 2007, in an editorial published in Critical Care Medicine, Dr. Parker claimed that better methods for determining cardiac preload and cardiac performance are badly needed to guide the clinician in the management of our critically ill patients, but until these methods become more widely available we are left with pressure measurements and clinical judgment [29]. This seems to still be the case for many anesthesiologists, even though new technologies and devices, including those that monitor dynamic predictors of fluid responsiveness, are indeed widely available.

### Study limitations

The accuracy of our survey can be impacted by ascertainment and non response bias. Our response rate was relatively small, and the clinicians who responded may not be representative, which could impact the external validity of these results. This is a common limitation to online or email surveys, and has been well documented [30,31]. One way of bypassing this limitation is to use a professional mailing list in order to reach a specific target population [31] as we did in the present study. However, it is interesting to observe the concordance between ESA and ASA respondents' answers and our results seem in accordance with commonly observed practice in Europe and in the US. Moreover, our goal was not to develop recommendations based on the extent of existing practice, but rather to establish a baseline against which consensus proposals would be developed. We also believe these results can be used in the future to assess the effects of guidelines/recommendations on clinical practice and hemodynamic monitoring/management in patients undergoing high risk surgery.

## Conclusions

In conclusion, these results point to a considerable gap between the accumulating evidence about the benefits of perioperative hemodynamic optimization and the available technologies that may facilitate its clinical implementation, and clinical practices in both Europe and the US. In addition, clinical practice may be heavily influenced by local factors that may not be justified by basic physiological considerations and the recently published body of evidence.

We postulate that we may be missing an enormous opportunity for better hemodynamic understanding, management and standardization. Clinicians may have adequate physiological knowledge but clinical application needs to be improved. Better communication, exchange of ideas and exposure to new technologies may decrease the observed differences between American and European anesthesiologists. On the local level, practical bedside teaching, simulation, and well-defined workshops may help to promote the use of appropriate hemodynamic management and goal directed therapy concepts. Recent and most important advances in anesthesia and critical care have been provided by "check list" implementations [32,33]. Standardization of practice and making sure that adequate therapies are delivered effectively may be the next step for hemodynamic management of patients undergoing high-risk surgery.

## Key messages

• Our results point to a considerable gap between the accumulating evidence about the benefits of perioperative hemodynamic optimization and the available technologies that may facilitate its clinical implementation, and clinical practices in both Europe and the US.

• Clinical practice may be heavily influenced by local factors that may not be justified by basic physiological considerations and the recently published body of evidence. We may thus be missing an enormous opportunity for better hemodynamic understanding, management and standardization.

• This lack of application of goal directed therapy concepts is not related to a lack of knowledge.

• The results of this email survey with a low response rate seems to emphasize the need for large clinical studies in order to demonstrate the clinical utility of intraoperative goal directed therapy during high risk surgery.

## Abbreviations

ASA: American Society of Anesthesiologists; CO: cardiac output; CVP: central venous pressure; DO2: oxygen delivery; ESA: European Society of Anaesthesiology; PAC: pulmonary artery catheter

## Competing interests

Maxime Cannesson is a consultant for Edwards Lifesciences (USA), Covidien (USA), Masimo Corp. (USA), ConMed (USA), Philips Medical System (Germany), CNsystem (Austria), BMeye (Netherlands), and Fresenius Kabi (Germany). Gunther Pestel is a consultant for BMeye (Netherlands) and Fresenius Kabi (Germany). Cameron Ricks has no conflict of interest to declare. Andreas Hoeft is a consultant for Edwards Lifesciences (USA). Azriel Perel is a consultant for BMeye (Netherlands), Pulsion (Germany), and FlowSense (Israel).

## Authors' contributions

MC designed the study, collected and analyzed the data, drafted the manuscript, and gave final approval of the manuscript. GP designed the study, analyzed the data, drafted the manuscript, and gave final approval of the manuscript. CR collected the data and gave final approval of the manuscript. AH analyzed the data, drafted the manuscript, and gave final approval of the manuscript. AP designed the study, analyzed the data, drafted the manuscript and gave final approval of the manuscript.

All authors read and approved the final version of the manuscript.

## Supplementary Material

Additional file 1 **Appendix 1**. Full questionnaire sent to American Society of Anesthesiologists members. A pdf. file with the full length questionnaire. Click here for file

Additional file 2 **Appendix 1**. Full questionnaire sent to the European Society of Anaesthesiology members. A pdf. file with the full length questionnaire. Click here for file
