# Supplementary material for: Hemodynamic monitoring and management in patients undergoing high risk surgery: a survey among North American and European anesthesiologists
Source: Crit Care. 2011 Aug 15;15(4):R197. doi: 10.1186/cc10364 (PMC3387639; doi:10.1186/cc10364)
Supplement: Additional file 2 — Appendix 1. Full questionnaire sent to the European Society of Anaesthesiology members. A pdf. file with the full length questionnaire. [file cc10364-S2.PDF]

ESA Hemodynamic Monitoring Questionnaire

1. Hemodynamic Management in Patients Undergoing High Risk Surgery

For the following questionnaire, we will define high risk surgery patients as patients aged 18 years or older presenting for major surgery expected to last more than 1.5 hours and having at least two of the following criteria:
1. Cardiac or respiratory illness resulting in functional limitation
2. Extensive surgery planned for carcinoma involving bowel anastamosis
3. Predictable acute massive blood loss (>2.5 litres)
4. Aged over 70 years with functional limitation of one or more organ systems
5. Septicemia (positive blood cultures or septic focus)
6. Respiratory failure (PaO_2 < 8 kPa on FiO_2 >0.4 i.e. PaO_2:FiO_2 ratio < 20 kPa or ventilation >48hours)
7. Acute abdominal catastrophe (e.g. pancreatitis, perforated viscous, gastro-intestinal bleed)
8. Acute renal failure (urea >20 mmol l-1, creatinine >260 µ mol l-1)
9. Surgery for abdominal aortic aneurysm
10. Disseminated malignancy

1. If you do not provide or directly supervise anesthesia for this type of patient, please indicate so by filling in the box below. Thank you.

- I do not provide or directly supervise anesthesia for this type of patient.
- I provide or directly supervise anesthesia for this type of patient.

2. Hemodynamic Management in Patients Undergoing High Risk Surgery

2. How many times in a typical work week do you provide or directly supervise anesthesia for a high risk surgery patient?

- Rarely or Never
- 1 to 5 times a week
- 6 to 10 times a week
- More than 11 times a week

3. Hemodynamic Management in Patients Undergoing High Risk Surgery

3. Which statement best describes your practice setting?

- University Hospital
- General Hospital
- Private Practice
- Other

Other (please specify)

4. Hemodynamic Management in Patients Undergoing High Risk Surgery

4. Does your institution or group have a written protocol, care guide, or statement concerning hemodynamic management in this setting?

- Yes
- No
- Unsure or don’t know

5. Hemodynamic Management in Patients Undergoing High Risk Surgery

5. What hemodynamic monitoring do you routinely use for the management of high risk surgery patients? (please, mark all that apply)

- Central venous pressure
- Cardiac ouput
- Pulmonary capillary wedge pressure
- Central venous saturation (SvO2)
- Stroke Volume Variation
- Systolic Pressure Variation
- Pulse Pressure Variation
- Mixed venous saturation (ScvO2)
- Non-invasive arterial pressure
- Transesophageal echocardiography
- Global end diastolic volume
- Near infrared spectroscopy
- Oxygen delivery (DO2)
- Plethysmographic Waveform Variation
- Invasive arterial pressure

6. Hemodynamic Management in Patients Undergoing High Risk Surgery

6. How frequently do you try to optimize arterial pressure intraoperatively in this setting?

- Never
- Less than 5 percent of the time
- Between 6 and 25 percent of the time
- Between 26 and 50 percent of the time
- Between 51 and 75 percent of the time
- More than 75 percent of the time

7. Hemodynamic Management in Patients Undergoing High Risk Surgery

7. How frequently do you try to optimize central venous pressure in this setting?

- Never
- Less than 5 percent of the time
- Between 6 and 25 percent of the time
- Between 26 and 50 percent of the time
- Between 51 and 75 percent of the time
- More than 75 percent of the time

8. Hemodynamic Management in Patients Undergoing High Risk Surgery

8. How frequently do you try to optimize cardiac output in this setting?

- Never
- Less than 5 percent of the time
- Between 6 and 25 percent of the time
- Between 26 and 50 percent of the time
- Between 51 and 75 percent of the time
- More than 75 percent of the time

9. Hemodynamic Management in Patients Undergoing High Risk Surgery

9. How frequently do you try to optimize central venous oxygen saturation (SvO2) in this setting?

- Never
- Less than 5 percent of the time
- Between 6 and 25 percent of the time
- Between 26 and 50 percent of the time
- Between 51 and 75 percent of the time
- More than 75 percent of the time

10. Hemodynamic Management in Patients Undergoing High Risk Surgery

10. How frequently do you try to optimize mixed venous oxygen saturation (ScVO2) in this setting?

- Never
- Less than 5 percent of the time
- Between 6 and 25 percent of the time
- Between 26 and 50 percent of the time
- Between 51 and 75 percent of the time
- More than 75 percent of the time

11. Hemodynamic Management in Patients Undergoing High Risk Surgery

11. How frequently do you try to optimize dynamic parameters of fluid responsiveness (Pulse Pressure Variations, Systolic Pressure Variations, Plethysmographic Waveform Variations) in this setting?

- Never
- Less than 5 percent of the time
- Between 6 and 25 percent of the time
- Between 26 and 50 percent of the time
- Between 51 and 75 percent of the time
- More than 75 percent of the time

12. Hemodynamic Management in Patients Undergoing High Risk Surgery

12. If you optimize hemodynamics in your high risk surgery patients, when do you do it?

- 1. Before anesthesia induction
- 2. After anesthesia induction
- 3. During surgery
- 4. In the postoperative period

13. Hemodynamic Management in Patients Undergoing High Risk Surgery

13. When do you think that hemodynamic optimization is of most value?

- 1. Before anesthesia induction
- 2. After anesthesia induction
- 3. During surgery
- 4. In the postoperative period

14. Hemodynamic Management in Patients Undergoing High Risk Surgery

14. Regarding respiratory variations in arterial pulse and/or systolic pressure: how do you measure these indices in the clinical setting?

- 1. Eyeballing
- 2. Manual calculation
- 3. Automatic measurement using specific software

If you use automatic measurement using a dedicated software, please specify which one.

15. Hemodynamic Management in Patients Undergoing High Risk Surgery

15. What technique do you use to monitor cardiac output? (please, mark all that apply)

- Swan Ganz catheter
- LiDCO Monitor
- PiCCO Monitor
- Transesophageal echocardiography
- Thoracic bioimpedance
- Other
- Esophageal Doppler
- Vigileo Monitor

Other (please specify)

16. Hemodynamic Management in Patients Undergoing High Risk Surgery

16. If you do not monitor cardiac output routinely in these patients, what are the main reasons for not monitoring it? (please, mark all that apply)

- Cardiac output monitoring does not provide any additional clinically relevant information in this setting
- I use SvO2 and/or ScVO2 as surrogates for cardiac output monitoring
- I use dynamic parameters of fluid responsiveness (Pulse Pressure Variations, Systolic Pressure Variations, Plethysmographic Waveform Variations) as surrogates for cardiac output monitoring
- Available cardiac output monitoring solutions are unreliable
- Available cardiac output monitoring solutions are too invasive

17. Hemodynamic Management in Patients Undergoing High Risk Surgery

Reminder:

In this questionnaire we define high risk surgery patients as patients aged 18 years or older presenting for major surgery expected to last more than one and a half hours and presenting at least two of the following criteria:

1. Severe cardiac or respiratory illness resulting in severe functional limitation
2. Extensive surgery planned for carcinoma involving bowel anastamosis
3. Predictable acute massive blood loss (>2.5 litres)
4. Aged over 70 years with functional limitation of one or more organ systems
5. Septicaemia (positive blood cultures or septic focus)
6. Respiratory failure (PaO_2 < 8 kPa on FiO_2 >0.4 i.e. PaO_2:FiO_2 ratio < 20 kPa or ventilation >48hours)
7. Acute abdominal catastrophe (e.g. pancreatitis, perforated viscous, gastro-intestinal bleed)
8. Acute renal failure (urea >20 mmol l-1, creatinine >260 µ mol l-1)
9. Surgery for abdominal aortic aneurysm
10. Disseminated malignancy

17. What are your indicators for volume expansion in this setting (diagnostic tools)? (please, mark all that apply)

- Clinical experience
- Stroke Volume Variation
- Cardiac ouput
- Pulse Pressure Variation or Systolic Pressure Variation
- Urine output
- Pulmonary capillary wedge pressure
- Plethysmographic Waveform Variation
- Central venous pressure
- Central venous saturation (SvO2)
- Blood pressure
- Mixed venous saturation (ScvO2)
- Global end diastolic volume
- Transesophageal echocardiography

18. Hemodynamic Management in Patients Undergoing High Risk Surgery

18. How do you routinely assess the hemodynamic effects of volume expansion in this setting?

- Decrease in stroke volume variation
- Increase in cardiac output
- Decrease in plethysmographic waveform variation
- Increase in central venous saturation (SvO2)
- Decrease in pulse pressure variation or systolic pressure variation
- Decrease in heart rate
- Increase in urine output
- Increase in blood pressure
- Increase in mixed venous saturation (SvO2)

19. Hemodynamic Management in Patients Undergoing High Risk Surgery

19. In your opinion, what best predicts an increase in cardiac output following volume expansion?

- Blood pressure
- Pulse pressure variation or systolic pressure variation
- Central venous pressure
- Pulmonary capillary wedge pressure
- Mixed venous saturation (ScvO2)
- Stroke volume variation
- Central venous saturation (SvO2)
- Plethysmographic waveform variations
- Transesophageal echocardiography
- Global end diastolic volume
- Clinical experience
- Cardiac output

20. Hemodynamic Management in Patients Undergoing High Risk Surgery

20. What is your first choice solution for volume expansion?

- Hydroxyethylstarch solutions
- Blood derived products
- Human albumin
- Crystalloids
- Dextrans
- Gelatin

21. Hemodynamic Management in Patients Undergoing High Risk Surgery

21. Do you or your department/group manage these patients in the intensive care unit?

- Yes
- No

22. Hemodynamic Management in Patients Undergoing High Risk Surgery

22. If not, who manages these patients in the ICU?

- Critical Care physicians
- Surgeons
- Other anesthesiologists
- Other physicians than anesthesiologists or surgeons
- Mixed population

23. Hemodynamic Management in Patients Undergoing High Risk Surgery

23. Do you believe that oxygen delivery to the tissues is of major importance in patients during high risk surgery?

- Yes
- No

24. Hemodynamic Management in Patients Undergoing High Risk Surgery

24. What parameter(s) is (are) involved in oxygen delivery to the tissues?

- PaO2
- SaO2
- Central venous pressure
- Cardiac Output
- Hemoglobin
- Arterial Pressure

25. Hemodynamic Management in Patients Undergoing High Risk Surgery

25. Do you believe that your current hemodynamic management could be improved?

- Yes
- No

26. Hemodynamic Management in Patients Undergoing High Risk Surgery

26. Which statement best describes you?

- I am an anesthesiologist predominantly caring for transplant surgery patients
- I am an anesthesiologist predominantly caring for cardiac surgery patients
- I am an anesthesiologist predominantly caring for patients not having transplant or cardiac surgery
- I am an anesthesiologist predominantly practicing intensive care
- I am an anesthesiologist caring for a variety of patients
- Other

Other (please specify)

27. Hemodynamic Management in Patients Undergoing High Risk Surgery

27. Did your training include a fellowship year?

- Yes
- No
- Not applicable

28. Hemodynamic Management in Patients Undergoing High Risk Surgery

28. If yes, which one(s)?

- Critical care medicine
- Pediatric anesthesiology
- Pain
- Research
- Cardiac anesthesiology
- Other

Other (please specify)

29. Hemodynamic Management in Patients Undergoing High Risk Surgery

29. When did you finish your training?

- After the year 2000
- 1990-1999
- 1980-1989
- Prior to 1980

30. Hemodynamic Management in Patients Undergoing High Risk Surgery

30. How many intensive care unit beds does your primary hospital have?

- 7 or less
- 8 to 10
- 11 to 15
- 16 to 20
- 21 to 30
- 31 to 40
- More than 40

31. Hemodynamic Management in Patients Undergoing High Risk Surgery

31. How many beds does your primary hospital have?

- 100 or less
- 101 to 250
- 251 to 500
- 501 to 1,000
- More than 1,000

32. Hemodynamic Management in Patients Undergoing High Risk Surgery

32. What is the population of your practice location?

- Less than 10,000
- 10,000 to 50,000
- 50,001 to 100,000
- 100,001 to 500,000
- More than 500,000

33. Hemodynamic Management in Patients Undergoing High Risk Surgery

* **33. In which country is your practice?**

34. Hemodynamic Management in Patients Undergoing High Risk Surgery

Thank you very much for taking time to answer these questions.
